# The B-Cell-Specific Ablation of B4GALT1 Reduces Cancer Formation and Reverses the Changes in Serum IgG Glycans during the Induction of Mouse Hepatocellular Carcinoma

**DOI:** 10.3390/cancers14051333

**Published:** 2022-03-04

**Authors:** Jichen Sha, Rongrong Zhang, Jiteng Fan, Yong Gu, Yiqing Pan, Jing Han, Xiaoyan Xu, Shifang Ren, Jianxin Gu

**Affiliations:** NHC Key Laboratory of Glycoconjugates Research, Department of Biochemistry and Molecular Biology, School of Basic Medical Sciences, Fudan University, 130 Dongan Road, Shanghai 200032, China; 19111010018@fudan.edu.cn (J.S.); 19211010018@fudan.edu.cn (R.Z.); 20211010020@fudan.edu.cn (J.F.); 20111010013@fudan.edu.cn (Y.G.); 18211010019@fudan.edu.cn (Y.P.); 17111010036@fudan.edu.cn (J.H.); 18111010037@fudan.edu.cn (X.X.)

**Keywords:** hepatocellular carcinoma, immunoglobulin G, glycan, β1,4-galactosyltransferase 1, mouse

## Abstract

**Simple Summary:**

As serum IgG glycosylation is associated with various cancers, our goal is to explore whether serum IgG galactosylation and its associated glycans could be used as tumor markers associated with hepatocellular carcinoma (HCC). At the same time, we explore the effect of the B-cell-specific ablation of B4GALT1 on HCC and finally analyze whether the low incidence of female cancer was related to the findings from the above perspective. The results demonstrate that the tumor marker of serum IgG glycosylation is galactosylation and its associated glycans and that the B-cell-specific ablation of B4GALT1 reduces HCC formation by reducing serum IgG galactosylation levels and by modulating the associated glycans, meaning that the lower incidence of cancer in women may be related to minor changes in the B-cell B4GALT1 and unchanged serum IgG galactosylation levels. This study aims to provide a theoretical basis for the early diagnosis and prevention of HCC and to determine why it has such a high incidence in males.

**Abstract:**

Serum immunoglobulin G (IgG) glycosylation, especially galactosylation, has been found to be related to a variety of tumors, including hepatocellular carcinoma (HCC). However, whether IgG glycan changes occur in the early stages of HCC formation remains unclear. We found that the galactosylation level increased and that the related individual glycans showed regular changes over the course of HCC induction. Then, the effect of the B-cell-specific ablation of β1,4galactosyltransferase 1 (CKO B4GALT1) and B4GALT1 defects on the IgG glycans that were modified during the model induction process and HCC formation is investigated in this study. CKO B4GALT1 reduces serum IgG galactosylation levels and reduces cancer formation. Furthermore, insignificant changes in the B-cell B4GALT1 and unchanged serum IgG galactosylation levels were found during cancer induction in female mice, which might contribute to the lower cancer incidence in female mice than in male mice. The gender differences observed during glycan and B4GALT1 modification also add more evidence that the B4GALT1 in B cells and in serum IgG galactosylation may play an important role in HCC. Therefore, the findings of the present research can be used to determine the methods for the early detection of HCC as well as for prevention.

## 1. Introduction

Hepatocellular carcinoma is an extremely serious tumor with a 5-year survival rate, according to the duplication report, of only 18% [1]. The incidence of HCC has continued to increase and ranks as the second leading cause of cancer-related deaths worldwide [2]. Although HCC patients improved after surgery, chemotherapy and radiotherapy [3], the 5-year survival rate is still not optimistic due to the lack of timely diagnosis, recurrence and tumor metastasis. Thus, there is an urgent need to explore more accurate early diagnosis methods and clearer mechanisms. Understanding the molecular events driving HCC is of paramount importance for the discovery of biomarkers for effective diagnosis or treatment [4].

Glycosylation is one of common protein post-translational modifications [5]. Glycans are one of the main regulators of biological processes and are involved in the control of protein folding [6]. Immunoglobulin G (IgG), highly abundant in serum, is a glycosylated protein and is responsible for most cancer immune surveillance functions [7]. Our previous study found that IgG galactosylation has great potential as a biomarker for cancer, including HCC [8]. Thus, it is very important for verifying the potential of IgG glycans as biomarkers for early diagnosis or treatment options. 

Contrary to the complex genetic backgrounds and environments that are associated with humans, mice have a unified breeding environment. C57BL/6 mice are the classic objects for cancer research. Therefore, changes in the characteristics of IgG glycans during HCC progression can be dynamically and continuously monitored in mice models. Exploring the changes in serum IgG glycans during the induction and development of mouse liver cancer allows IgG glycan change patterns to be revealed and also allows us to understand the possible role of IgG glycosylation in HCC. 

In this study, we first established a classic mouse HCC model induced by DEN/CCl_4_ [9]. Generally speaking, studies using mice models typically begin 8 months after DEN administration (6 months after DEN/CCl_4_ administration) because obvious tumors tend to have formed at this point. However, it is difficult to determine mechanisms for early diagnosis using this methodology. Studying the HCC induction, including its early stages, might favor the discovery of early diagnosis biomarkers as well as the related molecules that drive HCC, neither of which have been reported on in detail [4,10,11]. The screening of mouse serum IgG tumor markers is of great significance for early stage cancer research. In this study, the serum IgG glycans at the four time points over the entire DEN/CCl_4_ induction course were investigated, including at 2 months, 3 months, 8 months, and 10 months after DEN injection (0 months, 1 month, 6 months, and 8 months after DEN/CCl_4_ administration). β1,4-galactosyltransferase 1 (B4GALT1) is a type II transmembrane glycoprotein that resides in the Golgi apparatus of higher eukaryotic cells. It can catalyze the formation of β1,4 glycosidic bonds and can promote protein galactosylation [12]. Yuanyan Wei et al. found that B4GALT1 can be regulated by HBx and that it promotes the development of liver cancer cells [13]. The B lymphocyte is the major source of serum IgG [14]. Considering the relationship of the IgG glycans with spleen B cells, the expression of B4GALT1 in spleen B cells is specially investigated in the present research. In addition, its role in B4GALT1 on HCC formation and its association with HCC-related IgG glycan alteration are further explored by performing cancer induction in CD19-cre^+/−^ B4GALT1^flox/flox^ mice and in B4GALT1-deficient (B4GALT1^+/−^) mice. 

In addition, it is well known that men are more likely to develop HCC than women, a finding that has been confirmed in mouse HCC models [15]. Are there similar gender differences in terms of IgG glycan alteration in DEN/CCl_4_-induced mouse HCC models? The same administration regimen is performed on female mice to observe whether the gender differences in mouse HCC are also related to the changes in IgG glycan and B cell B4GALT1 expression, which might provide clues to the underlying mechanism of gender differences in HCC incidence.

This is the first time that serum IgG glycan changes at multiple time points in a DEN/CCl_4_-induced mouse HCC model have been explored. The effect of the B-cell-specific ablation of B4GALT1 and B4GALT1 defects IgG glycans modified during the model induction process is explored. Additionally, whether the B-cell-specific ablation of B4GALT1 has an effect on HCC or whether HCC promotes the expression of B4GALT1 in B cells is investigated, indirectly indicating a relationship between serum IgG galactosylation and HCC. Additionally, whether the gender differences observed in mouse HCC incidence are also related to the changes in IgG glycan and B4GALT1 expression in the B cells is studied.

## 2. Materials and Methods

### 2.1. Mice and Management

All animal procedures were conducted and approved in accordance with the guidelines of the Ethical Committee for Animal Experiments (Fudan University, Shanghai, China). Healthy two-week-old wild-type (WT) C57BL/6 mice, two-week-old CD19-cre^+/−^ B4GALT1^flox/flox^ C57BL/6 mice, and two-week-old B4GALT1^+/−^ C57BL/6 mice were used in this research. Both male and female mice were randomly divided into five groups treated as follows: control(C), solvent (vehicle, V), WT mice with DEN/CCl_4_ administration (H), CD19-cre^+/−^ B4GALT1^flox/flox^ mice with DEN/CCl_4_ administration (K), and B4GALT1^+/−^ mice with DEN/CCl_4_ administration (HK). No treatment was administered to the control group. For group V, WT mice were injected intraperitoneally with 50 mg/kg of oleyl tricaprylate (Sigma-Aldrich, St. Louis, MO, USA) at two weeks of age and with 0.5 mg/kg of corn oil (Aladdin, Shanghai, China) at 1 month of age. Corn oil was injected once a week for six weeks. For group H, WT mice were injected intraperitoneally with 50 mg/kg of diethylnitrosamine (DEN, Sigma-Aldrich, St. Louis, MO, USA, dissolved tricaprylin) at two weeks of age and with 0.5 mg/kg of CCl_4_ (Sinopharm Chemical Reagentcorn Co., Ltd., Shanghai, China, oil dissolves) at one month of age. CCl_4_ was injected once a week for six weeks. For group K, CD19-cre^+/−^ B4GALT1^flox/flox^ C57BL/6 mice were administered the same regime as the one outlined for group H. For group HK, B4GALT1^+/−^ C57BL/6 mice were administered the same regime at the one outlined for group H. Female WT C57BL/6 mice were also investigated in this research. The treatment protocols for the female groups were the same as those administered to the male mice. A total of four time points across the whole DEN/CCl_4_ induction course were investigated: 2 months, 3 months, 8 months, and 10 months after DEN injection (0 months, 1 month, 6 months, and 8 months after DEN/CCl_4_ administration). The mice were sacrificed, and the livers of the mice were frozen and stored in a refrigerator (Haier, Qingdao, China) at −80 °C for later use.

### 2.2. Serum Collection and Weight Measurement

Blood was taken at 0 months, 1 month, 6 months, and 8 months from the eye socket after DEN/CCl_4_ administration. Body weight was monitored during cancer induction, after DEN administration, and after each induction of CCl_4_, creating a total of seven time points. 

### 2.3. Histopathological Analysis

The removed mouse liver tissue was immersed in 4% paraformaldehyde, embedded in paraffin after being fixed for a period of time, and cut into 5–6 μm paraffin sections with a microtome (Leica, Shanghai, China). The obtained paraffin sections were stained with hematoxylin and eosin (HE), and finally the whole field of view was scanned by Nikon optical microscope (Nikon, Tokyo, Japan) and the field of view was photographed under a 200× microscope. The calculation method of the incidence of cancer is to count the number of mice in group H whose liver tissue has obvious tumor formation under HE. For example, 1 month after administration, we dissected 5 WT male mice, and no mice developed tumors. The incidence of cancer was 0. Then, 6 months after administration, we dissected 5 male WT mice, 4 mice had obvious tumor formation by HE staining, and 1 had no evident change. We calculated that the cancer incidence rate was 80%.

### 2.4. Isolation of Spleen B Cells

Take mouse spleen, cut 1/3 of it into a small plate, transfer it to a cell sieve, put the cell sieve into a small plate, completely chop the tissue, grind the chopped spleen, add Hack’s reagent and fully grind it until sediment is no longer visible by the naked eye. Transfer the cell fluid to a 50 mL centrifuge tube, add Hack’s reagent, rinse the remaining solids in the plate thoroughly and pour it into a 50 mL centrifuge tube, centrifuge at 300× *g* for 3 min, discard the supernatant, add 1 ml of Hack’s to dissolve again, and transfer to 15 mL centrifuge tube, fill up with red blood cell (RBC) lysis buffer (BD Pharmingen, San Diego, CA, USA) in the dark, shake on a shaker at 300 rpm for 20 min, centrifuge at 300× *g* for 3 min, discard the supernatant, add 500 μL Hack’s reagent, centrifuge at 300× *g* for 3 min, add the mixed solution buffer 500 μL, then add 10 μL of CD19 microbeads (Miltenyi Biotec, Bergisch Gladbach, Germany), after 15 min incubation, add about 500 μL buffer to mix well, at the same time balance about 3 mL of BSA mixed buffer with a balancer, centrifuge at 300× *g* for 3 min, discard the supernatant, and add 500 μL buffer to mix. uniform. Add 3 mL of buffer to the balancer (Miltenyi Biotec, Bergisch Gladbach, Germany), pour in the liquid, add 500 μL buffer, change the tube and press in.

### 2.5. RNA Extraction, Reverse Transcription and Quantitative Real-Time PCR

The RNA extraction procedure refers to the user guide of EZBioscience Universal RNA Purification(EZBioscience, Roseville, CA, USA); the instruction number is EZB-RN4. The reverse transcription step refers to the instruction manual of EZBioscience 4 × EZscript Reverse Transcription Mix II (with gDNA Remover) (EZB-RT2GQ, EZBioscience, Roseville, CA, USA). Although the first two parts can refer to the methods in the manual, for the final quantitative real-time PCR, each person’s processing method is different, and we describe our method in detail. First, the primers of B4GALT1 were slowly dissolved at room temperature, and 2 × SYBR Green qPCR Master Mix (ROX 1) (EZBioscience, Roseville, CA, USA) was prepared in advance to dissolve slowly at room temperature. After dissolution, the temperature was kept on ice. The primers of B4GALT1 were calculated according to the maximum number of samples required. A total of 0.4 μL of upstream and downstream primers for each sample and 5.2 μL of ultrapure water for each sample in advance were mixed, then add 6 μL to each well of 96PCR plate, and finally, add reverse reagents to each well. A total of 4 μL of the 10-fold diluted sample after transcription was added, and finally 10 μL of 2 × SYBR Green qPCR Master Mix (ROX 1) was added to each well, and qPCR was performed after rotating and mixing. The obtained CT values were obtained using the 2^(−ΔΔCt)^ calculation method to obtain the relative value of the gene.

### 2.6. Purification of IgG N–Glycan Chains

The filter was added to the deep well plate with tweezers. Protein G (Bestchrom, Shanghai, China) should be diluted twice with 20% ethanol, and 100 μL of the dilution should be added to each well. The sample was washed two times with 400 μL 1 × PBS. In an ordinary 96-well plate, 40 μL of PBS and 20 μL of serum were added, the sample was transferred to the 96-well plate, and incubated with a film at room temperature for half an hour. It was centrifuged once, washed three times with 400 μL of PBS, washed three times with 200 μL of water, the lower layer was replaced with a new square 96-well plate, 100 μL of formic acid (FA, Merck, Darmstadt, Germany) was added and incubated for 10 min. After incubation, the sample was centrifuged and the samples were recovered to a new tip 96-well plate; the upper deep-well plate was washed with 100 μL of formic acid, washed three times with PBS, 200 μL of PBS was added to wrap the plate and then wrapped with tin foil. Finally, it was spin dried with a vacuum concentrator (Eppendorf, Hamburg, Germany) for 2 h.

### 2.7. IgG N–Glycan Enzymatic Hydrolysis and Labeling

After spin-drying, add 5 μL of water and 10 μL of 2% sodium dodecyl sulfate (SDS, Sigma Aldrich, St. Louis, MO, USA) to mix and denature, shake, and denature at 60 °C and 350 rpm for 10 min. Then, add 5 μL of 5 × PBS, add 5 μL of 4% NP40 (New England Biolabs, New York City, NY, USA), and add 1 μL of glycosidase (10-fold dilution, New England Biolabs, NY, USA) overnight at 37 °C. On the next day, 5 μL of 2-aminobenzamide (2-AB, Sigma Aldrich, St. Louis, MO, USA) was added to each sample and incubated at 60 °C for 2 h.

### 2.8. Purification of Labeled IgG N–Glycans

After the sample plate was taken out, it was centrifuged immediately, and 300 μL of 100% HPLC-grade acetonitrile (ACN; Merck, Darmstadt, Germany) was added. An amount of 200 μL 20 times-diluted sepharose solution was added to the filter plate. The plate was washed once with 200 μL 20% ethanol, twice with 200 μL Milli-Q water, and twice with 200 μL 96% ACN. The samples were transferred to a 96-well plate with a PVDF membrane and were incubated at 550 rpm for 10 min at room temperature. They were then centrifuged, and the waste was discarded at the end. The plate was washed 5 times with 200 μL 96% ACN. Finally, the IgG glycans were eluted with 200 μL Milli-Q water.

### 2.9. UPLC-FLR

For detailed steps, please refer to the Supplementary Material [16].

### 2.10. Statistical Analysis

Statistical analysis was performed using IBM SPSS Statistics Data Editor version 23.0 software (SPSS, Chicago, IL, USA). All data are presented as mean ± SD. The independent samples *t*-test was used for comparative analysis between two groups, and three groups or more were analyzed using one-way analysis of variance (ANOVA) and Tukey’s post hoc test. *p* < 0.05 was considered statistically significant.

## 3. Results

### 3.1. DEN/CCl_4_-Induced HCC Model Was Successfully Established, and Tumor Formation Was Obvious after 6 Months of Administration

**Figure 1:**
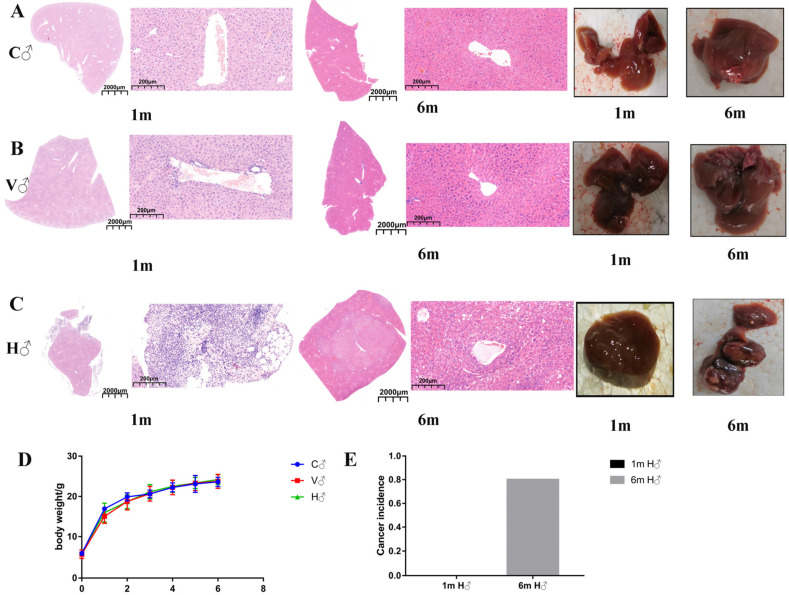
DEN/CCl_4_-induced HCC model was successfully established in male mice, and tumor formation was obvious after 6 months of administration. (**A**) Both representative total appearance and HE staining in group C showed no lesions at 1 month or at 6 months. (**B**) Both representative total appearance and HE staining in group V showed no lesions at 1 month or at 6 months after administration. (**C**) Both representative total appearance and HE staining in group H showed developed vacuolar degeneration, but no tumor formation at 1 month after administration, and both representative total appearance and HE staining showed obvious tumor formation at 6 months after administration. Hematoxylin and eosin; full scan image (×1.0), bars = 2000 μm; single-field image (×200), bars = 200 μm. (**D**) No differences were observed between the different groups in terms of body weight after CCl_4_ administration. (**E**) Cancer incidence was 0% within 1 month after administration and 80% within 6 months after administration. (C—control WT group; V—solvent WT group; H—HCC WT group; 1 m/6 m—1 month/6 months after administration; ♂—male mice).

Confirming the success of the HCC model is a critical step. At 1 month after administration (1 m), HE staining was performed. It was found that the histology of the WT blank control group (C) and WT solvent control group (V) were normal. Although the WT mice with DEN/CCl_4_ administration group (H) had evident vacuolar degeneration, no evident cancerous sites were found. Histopathological analyses were performed again 6 months after administration (6 m). Groups 6m C and V showed normal results. Since no differences were observed in the liver tissue samples between groups C and V, they will no longer be distinguished from each other in the following research results. However, group 6m H showed obvious tumor nodules. Both the representative total appearance and HE staining revealed that the liver structure was disordered and that the observed vacuolar degeneration was serious (Figure 1A–C). The liver is part of the digestive system. By monitoring the weight after DEN treatment and each cycle of CCl_4_ administration, it was found that there were no significant changes in body weight, which confirmed that there was no relationship between medication and body weight during the model period (Figure 1D). At 1 month after administration, cancer incidence was 0, but it was 80% 6 months after administration (Figure 1E). 

### 3.2. The Relative Abundance of Galactosylation and Some IgG glycans Is Highly Correlated with HCC

**Figure 2:**
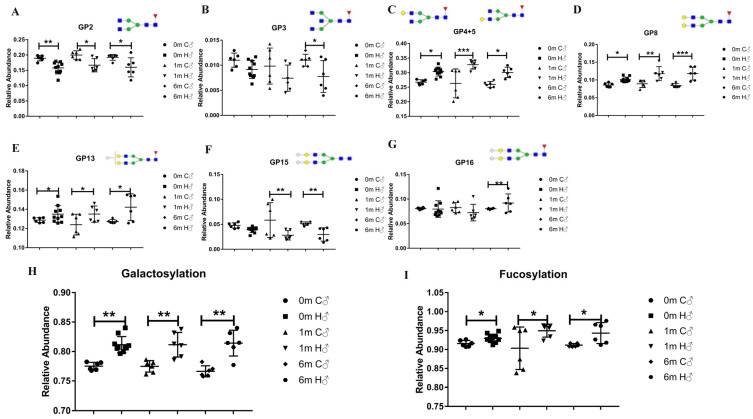
The changes in the serum IgG glycans between groups H and C at the three time points of 0, 1, and 6 months after administration during the cancer induction process in male mice, which indicated the relative abundance of galactosylation and that some IgG-N glycans are highly correlated with HCC. (**A**) GP2 in group H was lower than it was in group C at all three time points. (**B**) GP3 only decreased in group H at 6 months. (**C**) The sum of the relative abundances of GP4 and GP5 in group H was higher than it was in group C at all three time points. (**D**) GP8 in group H was higher than it was in group C at all three time points. (**E**) GP13 in group H was higher than in was in group C at all three time points. (**F**) GP15 decreased at 1 and 6 months. (**G**) GP16 only increased at 6 months. (**H**) Galactosylation in group H was higher than it was in group C at all three time points. (**I**) Fucosylation in group H was higher than it was in group C at all three time points. Structural symbols: blue square: N-acetylglucosamine; green circle: mannose; red triangle: fucose; yellow circle: galactose; white diamond: N-glycolylneuraminic acid. Data are expressed as mean ± SD. * *p* < 0.05, ** *p* <0.01, *** *p* <0.001. (C—control WT group; H—HCC WT group; 0 m/1 m/6 m—0 month/1 month/6 months after administration; ♂—male mice).

Previous studies have mentioned that serum IgG glycans are closely related to cancer; however, how the IgG glycans change throughout the HCC formation process remains unknown. After confirming the successful HCC induction, we began to explore the changes in serum IgG glycans at multiple time points during the cancer induction process in mice. We first analyzed 16 glycan UPLC peaks characterized in our previous work [17] at different time points in detail during the HCC induction process. The IgG glycan components and structure corresponding to the 16 glycan peaks are shown in Figure S1. As shown in Figure 2A–G, eight glycans were found to have been altered during HCC induction, including GP2, 3, 4, 5, 8, 13, 15, and 16. Among them, three glycans were decreased in group H, and they were found to have no terminal galactose (GP2 and 3) or no core fucose (GP15), while the other five glycans in group H that were shown to have increased are all fucosylated with a terminal galactose or sialic acid. Considering the fact that GP4 and GP5 are isomers with little overlap in chromatography and because they show similar change trends, we added them together in the following experiments to achieve better accuracy. When considering these IgG glycans over the course of HCC progression, it can be seen that some glycans changed at all three investigated time points, including GP2, 4, 5, 8, and 13, while others only changed at later stages in the induction process (Figure 2 and Table 1). All of the changes observed in the glycans reflect the increased levels of the galactosylation and fucosylation of the glycans in group H (Figure 2H,I). Both of them showed steady changes at the three time points; however, the galactosylation changes are more prominent (Figure 2 and Table 1). All of the above results indicate that the galactosylation of serum IgG and the related glycans were highly correlated with HCC, which is the focus of the following sections. The letters in Table 1 can be explained as follows: A, N-acetylglucosamine; B, bisecting structure; F, fucose; G, galactose; M, mannose; S, sialic acid.

### 3.3. The mRNA Level of B4GALT1 in Splenic B Cell Significantly Increased after Cancer Induction, and B-Cell-Specific Ablation of B4GALT1 Reduced Tumor Formation

**Figure 3:**
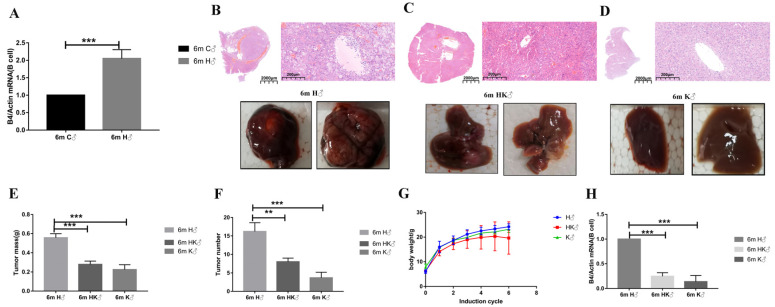
The mRNA level of B4GALT1 in splenic B cells significantly increased after cancer induction, and B-cell-specific ablation of B4GALT1 reduced tumor growth. (**A**) A total of 6 months after administration, the B4GALT1 mRNA level of spleen B cells significantly increased. (**B**) Both representative total appearance and HE staining showed obvious tumor formation in WT mice 6 months after administration. (**C**) Both representative total appearance and HE staining showed that tumors were suppressed in B4GALT1-deficient mice 6 months after administration. (**D**) Both representative total appearance and HE staining showed that the tumors from CD19-cre B4GALT1-floxed mice were suppressed 6 months after administration. Hematoxylin and eosin; full scan image (×1.0), bars = 2000 μm; single-field image (×200), bars = 200 μm. (**E**) Tumor mass statistics for group H, group HK, and group K. (**F**) Tumor number statistics for group H, group HK, and group K. (**G**) Body weight trend statistic changes during CCl_4_ induction process for group H, group HK, and group K. (**H**) The B4GALT1 mRNA level in the spleen B cells of CD19-cre B4GALT1-floxed mice and B4GALT1-deficient mice is indeed reduced. Data are expressed as mean ±SD. ** *p* < 0.01, *** *p* < 0.001. (C—control WT group; H—HCC WT group; HK—HCC B4GALT1^+/−^ group; K—HCC CD19-cre^+/−^ B4GALT1^flox/flox^ group; 1 m/6 m—1 month/6 months after administration; ♂—male mice).

Since the relative abundance of galactosylation and related glycans changes steadily over time, we explored how the B4GALT1 in spleen B cells changes after cancer induction and found that the B4GALT1 in spleen B cells also increased after cancer induction (Figure 3A). Therefore, the above results attracted our interest regarding the role of the B-cell-specific ablation of B4GALT1 in HCC. Strictly speaking, the previous study only implanted SiB4GALT1 tumor cells in nude mice, and this was also the first time that the HCC phenotype was studied in B4GALT1-deficient mice. As no tumors were found at 1m, no knockout mice were used for the research at this time. The 6m H group showed obvious tumor nodules. As we mentioned above, the liver structure was disordered, and the vacuolar degeneration was serious. Both the full-scan image and the individual area showed obvious cancer lesions (Figure 1C and Figure 3B). Differently, B4GALT1-deficient mice and CD19-cre B4GALT1-floxed mice showed reduced tumor nodules and liver disorders after cancer induction (Figure 3C,D). The CD19-cre B4GALT1-floxed mice and B4GALT1-deficient mice developed much smaller tumors and showed less tumor burden than the WT mice (Figure 3E,F). Interestingly, after comparing the WT and two types of knockout mice, there continued to be no differences in body weight observed (Figure 3G). Additionally, the mRNA level of B4GALT1 in splenic B cells was measured again and was indeed determined to be significantly lower in group K and in group HK than it was in group H (Figure 3H). The results confirmed that the B4GALT1 gene was successfully knocked out in the CD19+ B cells. The results suggest that the B-cell-specific ablation of B4GALT1 plays an important role in HCC. Considering that B4GALT1^+/−^ mice still have B4GALT1 in B cells, we compared untreated B4GALT1^+/−^ mice (group CK) with groups C, H, and HK. This also indicated that knockout of B4GALT1 exerted a greater effect in HCC conditions than in control conditions. Furthermore, it can be seen that HCC does not significantly upregulate B4GALT1 in B4GALT1^+/−^ mouse B cells, which further suggests that B4GALT1 is highly correlated with HCC (Figure S2).

### 3.4. B-Cell-Specific Ablation of B4GALT1 Reduces the Level of Serum IgG Galactosylation in HCC and Reverses the Changes in Galactose-Related Glycans in HCC

**Figure 4:**
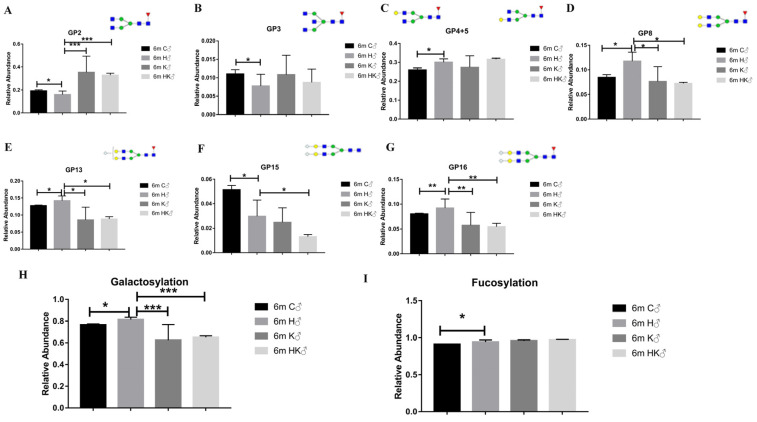
B-cell-specific ablation of B4GALT1 reduces the level of serum IgG galactosylation in HCC and reverses the changes induced in galactose-related glycans in HCC. (**A**) B-cell-specific ablation of B4GALT1 and systemic knockout of B4GALT1 both reversed the changes in the relative abundance of GP2. (**B**) B-cell-specific ablation of B4GALT1 and systemic knockout of B4GALT1 do not affect the relative abundance of GP3. (**C**) B-cell-specific ablation reverses the changes observed in the sum of the relative abundances of GP4 and GP5. (**D**) B-cell-specific ablation of B4GALT1 and systemic knockout of B4GALT1 both reversed the changes in the relative abundance of GP8. (**E**) B-cell-specific ablation of B4GALT1 and systemic knockout of B4GALT1 both reversed the changes in the relative abundance of GP13. (**F**) Systemic knockout of B4GALT1 promoted changes in the relative abundance of GP15. (**G**) B-cell-specific ablation of B4GALT1 and systemic knockout of B4GALT1 both reversed changes in the relative abundance of GP16. (**H**) B-cell-specific ablation of B4GALT1 and systemic knockout of B4GALT1 both reversed changes in the relative abundance of galactosylation. (**I**) B-cell-specific ablation of B4GALT1 and systemic knockout of B4GALT1 did not affect the relative abundance of fucosylation. Structural symbols are the same as Figure 2. Data are expressed as mean ± SD. * *p* < 0.05, ** *p* < 0.01, *** *p* < 0.001. (C—control WT group; H—HCC WT group; HK—HCC B4GALT1^+/−^ group; K—HCC CD19-cre^+/−^ B4GALT1^flox/flox^ group; 6 m—6 months after administration; ♂—male mice).

According to the above results, B4GALT1 was elevated in B cells in HCC, and the B-cell-specific ablation of B4GALT1 reduces cancer formation. We continued to explore the effect of the B-cell-specific ablation of B4GALT1 on the above-mentioned glycans that were shown to be altered in HCC. B4GALT1-deficient mice were compared with the CD19-cre B4GALT1-floxed mice to determine whether the galactosylation of serum IgG and the related glycans are affected by the B4GALT1 in the B cells. Among all of the glycans that were shown to be altered during HCC induction, the changes that were induced in four glycans were reversed in both group K and HK, including the agalactosylated glycan(GP2) and digalactosylated glycans (GP8, GP13, and GP16), all of which were fucosylated (Figure 4A,D,E,G). However, no statistical differences were observed in the changes in the agalactosylated glycan (GP3) and in the monogalactosylated glycan (GP4 + 5) in groups H and K as well as in groups H and HK (Figure 4B,C). The afucosylated digalactosylated glycan (GP15) not only did not reverse, but the relative abundance continued to decrease instead (Figure 4F). The galactosylation level was also affected by B4GALT1, but the fucosylation level was not affected by B4GALT1 (Figure 4H,I). Interestingly, there were no significant differences that were observed between the B4GALT1-deficient mice and the CD19-cre B4GALT1-floxed mice, indicating that serum IgG galactosylation is mainly regulated by B4GALT1 in B cells. This suggests that the B-cell-specific ablation of B4GALT1 reduces the level of serum IgG galactosylation in HCC and reverses the changes induced in the galactose-related glycans in HCC. 

### 3.5. DEN/CCl_4_-Induced IgG Galactosylation Alteration and Increased B-Cell B4GALT1 mRNA Levels in Male Mice Was Not Obviously Found in Female Mice

**Figure 5:**
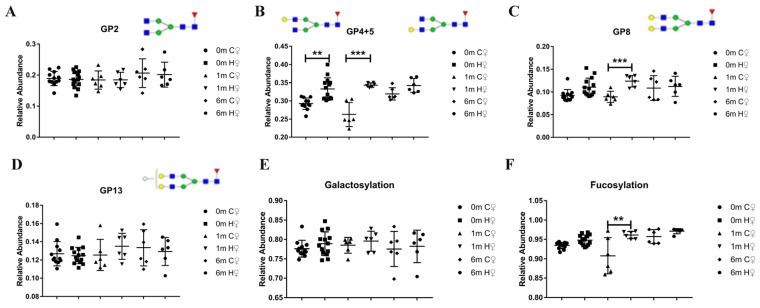
Female mice have a lower cancer incidence do not have the same stable IgG glycan changes as male mice, who have a higher high cancer incidence. (**A**) The relative abundance of GP2 in female mice does not change during the cancer induction process. (**B**) The sum of the relative abundances of GP4 and GP5 in female mice does not change regularly during the cancer induction process. (**C**) The relative abundance of GP8 in female mice only showed a decrease after the first month of the cancer induction process. (**D**) The relative abundance of GP13 in female mice did not change during the cancer induction process. (**E**) The relative abundance of galactosylation in female mice did not change during the cancer induction cases. (**F**) The relative abundance of fucosylation in female mice only showed a decrease one month after the initiation of the cancer induction process. Structural symbols are the same as Figure 2. Data are expressed as mean ± SD. ** *p* <0.01, *** *p* <0.001. (C—control WT group; H—HCC WT group; 0 m/1 m/6 m —0 month/1 month/6 months after administration; ♀—female mice).

**Figure 6:**
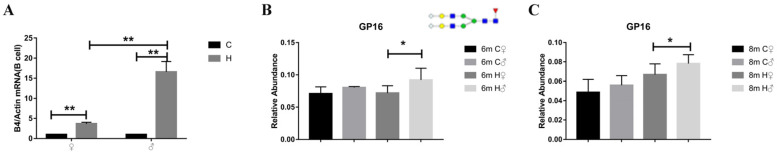
Female mice have a lower cancer incidence and do not show obvious increase in in B-cell B4GALT1 gene expression in contrast to male mice, who have a higher cancer incidence, and the relative abundance of GP16 is significantly lower in female mice than it is in male mice. (**A**) Approximately 1 month after administration, the B4GALT1 mRNA level in spleen B cells increased more significantly in males than in females. (**B**) The relative abundance of GP16 in males at 6 months after administration was significantly higher than that in females. (**C**) The relative abundance of GP16 in males at 8 months after administration was significantly higher than that in females.* *p* < 0.05, ** *p*< 0.01. (C—control WT group; H—HCC WT group; 6 m/8 m—6 months/8 months after administration; ♀—female mice; ♂—male mice).

We found that the level of galactosylation and B-cell B4GALT1 expression increased in the male mice HCC model, and that the B-cell-specific ablation of B4GALT1 inhibited the development of DEN/CCl_4_-induced HCC and reversed the changes observed in the galactosylation level. It is well known that men are more likely to develop HCC than women, and a similar gender disparity was found in the DEN-induced mouse HCC model [15]. Therefore, female mice were also treated with DEN/CCl_4_ in the same way that the male mice were to determine whether the gender differences in hepatocarcinogenesis in mice are also associated with the alterations observed for IgG glycan and B-cell B4GALT1 expression. First, we verified the low incidence of HCC in females when the female mice were treated with DEN/CCl_4_ in the same way as the male mice were (Figure S3). The serum IgG glycans of female mice are not as stable and regular as that of male mice (Figure S4). Then, we found glycans that showed regular changes at multiple time points during the cancer induction process in male mice were not found during the cancer induction process in female mice (Figure 5A–F). Notably, no obvious IgG galactosylation alterations were observed in the female model, which was not the case in the male mice model (Figure 5E). Changes in all serum IgG glycans during cancer induction in female mice are shown in Table S1. The B4GALT1 mRNA present in spleen B cells was also found lower in in the group H that comprised female mice compared to the group H that comprised male mice (Figure 6A). In addition, changes in glycan GP16 were only determined at time points where multiple HCCs had formed and were not found to have changed during the same time point in the female mouse model (Figure 6B). We further investigated the glycan GP16 level in the older DEN/CCl_4_-treated mice (8 months after DEN/CCl_4_). In the male mice model, GP16 remained altered in the same way as it appeared in the 6-month-old DEN/CCl_4_-treated mice, while it remained unchanged in female mice (Figure 6C). Because obvious tumors can usually be observed after 6 months, the present research does not focus on observations made at 8 months after administration, and these findings are only briefly described in the table in order to compare the different genders. The results indicated that changes in B4GALT1 mRNA and IgG glycans are related to a higher incidence of HCC in male mice. Additionally, these findings may hint at the underlying mechanism that accounts for this gender disparity in HCC incidence and can also be used to find new ways to prevent HCC in males.

## 4. Discussion

Conducting mouse HCC model research helps us to better understand human disease models in the future [18]. Since the serum IgG glycans change significantly during the natural aging process in mice, it is of little significance to compare group H over time, which was also the purpose of establishing a multi-time control group in this study. 

For the establishment of an HCC model using 2-week-old mice, the basis was also found in the literature [9,19]. Although there was an orthotopic mouse liver tumor model, which was quickly established [20], chemical induction was more traditional and reliable [21]. This model shows a transition from liver inflammation to liver cancer. It is indeed insufficient to emphasize that HCC ignores liver inflammation. Therefore, we will briefly discuss the possible relationship between IgG glycans and liver inflammation before carcinogenesis (0–1 month after DEN/CCl_4_ administration). We found that the HCC model basically had no effect on the weight changes that were observed. At the tissue level, the extent of liver cancer was determined by the tumor formation area [22]. 

In our previous work, the 16 serum IgG glycans of C57 mice had been characterized and confirmed [17]. GP2, GP4 + 5, and GP8 all showed regular trend changes at 0 months, 1 month, 6 months, and 8 months after DEN/CCl_4_ administration. Based on the results of this research, the galactosylation of IgG was out focus. At 8 months after administration, these four glycans did not change compared to the control group, indicating that the changes in the model are consistent in the early and mid-term and that the glycosylation level no longer changes at later periods. It is worth mentioning that GP16 did not change compared to group C at 0 months and the first month after administration, but these changes were significantly higher than those observed in the control group at the 6th and 8th months. This finding suggests that GP16 can be used as a serum tumor marker after tumorigenesis. It is well known that liver inflammation occurs in the early stages of this model [23]. We also found that the above-mentioned key IgG glycans (GP2, GP4 + 5, and GP8) also have a similar relationship with liver inflammation before HCC formation, that is, 0–1 month after DEN/CCl4 treatment. Interestingly, we found that the galactosylation of serum IgG and its related glycans were significantly changed before the HCC formation, so if we interfered with the galactosylation of IgG, would it affect the formation of HCC? If there is an effect, then it can be explained by the fact that IgG galactosylation leads to the formation of HCC rather than by the explanation that HCC formation promotes IgG galactosylation. 

The mRNA results for B4GALT1 in the splenic B cells indicate a significant increase in B4GALT1 in B cells during HCC. Summarizing the above results, CD19-cre B4GALT1-floxed mice and B4GALT1-deficient mice were used to study the effect of B4GALT1 on the glycans affected by HCC. In addition, the inflammatory response of B4GALT1-deficient mice is reduced, and the survival rate can be guaranteed during the breeding process [24]. However, other studies in the literature have mentioned growth retardation and the early death of B4GALT1 knockout mice and that their epithelial cells show proliferation and abnormal differentiation [25]. With reference to the literature and breeding experience [26], we decided to use B4GALT1^+/−^ mice for the experiments to prevent the possibility of death in homozygous mice. In our actual breeding process, B4GALT1^−/−^ mice have low yield and high homozygous lethality. So, we used B4GALT1^+/−^ mice instead of B4GALT1^−/−^ mice. 

The histopathological analysis revealed that the B-cell-specific ablation of B4GALT1 and B4GALT1 defects can reduces HCC formation. After analyzing the serum IgG glycan of the two kinds of knockout mice, it is not difficult to determine rules. Agalactosylated G0 (GP2) plays a role in the diagnosis of diseases [27]. First, for GP2, the relative abundance of two kinds of knockout mice was higher than that of WT mice. This shows that GP2 can be used as a serum tumor marker for the early monitoring of HCC. Monogalactosylated G1(GP4 + 5) also plays a role in the differential diagnosis of diseases [28]. However, B4GALT1 gene knockout did not significantly affect its relative abundance. This indicates that B4GALT1 gene knockout may change the galactosylation level of other proteins other than IgG, which affects the downregulation of the relative abundance of IgG-(GP4 + 5). Digalactosylated G2 (GP8) plays a role in disease treatment [29]. The relative abundance of GP8 in the two kinds of knockout mice was lower than that in the WT mice. This shows that GP8 can be used as a serum tumor marker for the early monitoring of HCC. Meanwhile, since serum IgG galactosylation levels are already elevated before HCC formation and because B-cell-specific ablation of B4GALT1 can reduce HCC formation, we believe that serum IgG galactosylation promotes HCC formation.

In addition to the four glycans related to the level of terminal galactosylation, GP13, which has two LacNAc with terminal sialic acid, also changed regularly at 0, 1, and 6 months. Similar to GP8, the relative abundance of GP13 of the two kinds of knockout mice were significantly lower than that of WT mice. After analyzing the glycan composition, it was not difficult to determine that both GP13 and GP8 have two galactose structures; the difference is that there is only one terminal sialic acid. In this paper, we guessed that the changing trends observed in the glycans on IgG with two galactose structures in serum were closely related to HCC. Both humans and mice showed a high correlation between IgG-G2 and HCC [30]. As mentioned above, GP16 began to increase significantly 6 months after administration, and the relative abundance of males was higher than that of females. A total of 6 months after administration, the B-cell-specific ablation of B4GALT1 and B4GALT1 defects significantly reduced the relative abundance of GP16. Combined with the changes in the HCC phenotype 6 months after administration, we once again confirmed that GP16 can be used as a serum marker for mouse HCC. Interestingly, the GP16 structure is similar to that of GP8 and GP13. They are all fucosylated biantennary glycans containing two LacNAc. These three IgG glycans showed regular changes at multiple time points in the WT mice and knockout mice HCC models. We did not think that this was a coincidence. Estrogen has been determined to be an in vivo regulator of IgG galactosylation in men and women [31]. However, no one has conducted in-depth research on IgG glycosylation between females, who have a lower cancer incidence, and males with a higher cancer incidence.

It was found that GP4 + 5, GP8, and GP13 did not change steadily at multiple time points as they did in the male mice. In addition, the relative abundance of serum IgG galactosylation did not change significantly in the female mice. After analyzing the B4GALT1 mRNA level in spleen B cells and the relative abundance of GP16, we found that there were more male mice with a significantly higher cancer incidence than there were female mice with a lower cancer incidence. Based on the premise of low cancer incidence in female mice, these results again verified that B4GALT1 and serum IgG galactosylation levels in B cells are highly correlated with HCC, although our study revealed the possibility that the incidence of male cancer is higher than that of females. Due to research limitations, there is no in-depth exploration of relevant mechanisms, such as the relationship between the expression of B4GALT1 in B cells and estrogen or androgen and the regulatory mechanism. The relationship between serum IgG galactosylation level and estrogen or androgen and the regulatory mechanism are worth exploring further, which will be of great help to the research on the incidence of clinical hepatocellular carcinoma in different genders. 

In summary, our results indicate that the serum IgG galactosylation level of male mice steadily increased in the HCC model. The relative abundance of GP2 steadily decreased in the HCC model. IgG-G2 (GP8 and GP13), which has two LacNAc structures, increased steadily in the model. B4GALT1 in the B cells of the spleen was indeed elevated after the induction of HCC. The B-cell-specific ablation of B4GALT1 and B4GALT1 deficiency can reduce HCC formation. At the same time, we found that the B-cell-specific ablation of B4GALT1 inhibited the increase in the IgG galactosylation serum level in mice. The relative increase in B4GALT1 in male mouse spleen B cells was also significantly higher than that observed in female mice. These may be related to the higher cancer incidence in males than in females. Our research revealed that the tumor markers of serum IgG glycosylation are galactosylation and its related glycans. Serum IgG galactosylation may promote HCC formation. B4GALT1 in B cells is elevated in HCC, and the B-cell-specific ablation of B4GALT1 reduces HCC formation possibly by reducing the level of serum IgG galactosylation and by regulating related glycans. The low cancer incidence in females may be related to the insignificant changes in B4GALT1 of B cell and the unchanged serum IgG galactosylation level. The above research provides a theoretical basis for clinical HCC intervention. However, more in-depth and more detailed mechanisms need to be studied.

## Figures and Tables

**Table 1:** The change trend of serum IgG glycan group H in male mice compared with group C at different time points in the process of cancer induction.

Glycan Peak	0 Month	1 Month	6 Month	8 Month	Component Name
GP1	-	-	-	↓	F(6)A1
GP2	↓↓	↓	↓	-	F(6)A2
GP3	-	-	↓	-	F(6)A2B
GP4 + 5	↑	↑↑↑	↑	-	F(6)A2G1
GP6	-	-	-	↓↓↓	F(6)A2[6]BG1
GP7	-	-	-	-	M6
GP8	↑	↑↑	↑↑↑	-	F(6)A2G2
GP9	-	-	-	-	F(6)A1G1S1
GP10	-	-	-	-	F(6)A2[6]G1S1
GP11	↓	-	-	-	F(6)A2[3]G1S1
GP12	-	-	-	-	A2G2S1
GP13	↑	↑	↑	-	F(6)A2G2S1
GP14	-	-	-	-	F(6)A2G2G(3)1S1
GP15		↓↓	↓↓	-	A2G2S2
GP16	-	-	↑↑	↑	F(6)A2G2S2
Galactosylation	↑↑	↑↑	↑↑	-	
Fucosylation	↑	↑	↑	-	
Sialylation	-	-	-	-	
Bisection	-	-	-	↓	
Monoantennary glycans	-	-	↓	-	

Footnote: ↑ and ↓ represent *p* < 0.05; ↑↑ and ↓↓ represent *p* < 0.01; ↑↑↑ and ↓↓↓ represent *p* < 0.001.

## Data Availability

The data presented in this study are available on request from the corresponding author.

## Supplementary Materials

The following supporting information can be downloaded at: https://www.mdpi.com/article/10.3390/cancers14051333/s1, Figure S1. Representative chromatogram of murine IgG N-glycans separated by UPLC-FLR after 2-AB labeling. Figure S2. DEN/CCl_4_ induced HCC model was successfully established, and tumor formation was obvious after 6 months of administration. Figure S3. mRNA levels of B4GALT1 in spleen B cells of four mice in group C, group CK, group H, and group HK. Figure S4. Female mice have no stable changes during the process of cancer induction. Method about UPLC-FLR. Table S1. The change trend of serum IgG N-glycan group H in female mice compared with group C at different time points in the process of cancer induction.
