# Supplementary material for: The B-Cell-Specific Ablation of B4GALT1 Reduces Cancer Formation and Reverses the Changes in Serum IgG Glycans during the Induction of Mouse Hepatocellular Carcinoma
Source: Cancers (Basel). 2022 Mar 4;14(5):1333. doi: 10.3390/cancers14051333 (PMC8909634; doi:10.3390/cancers14051333)
Supplement: Supplementary file 1 [file cancers-14-01333-s001.zip › cancers-1605514-supplementary.pdf]

Supplementary materials

<Chromatogram>

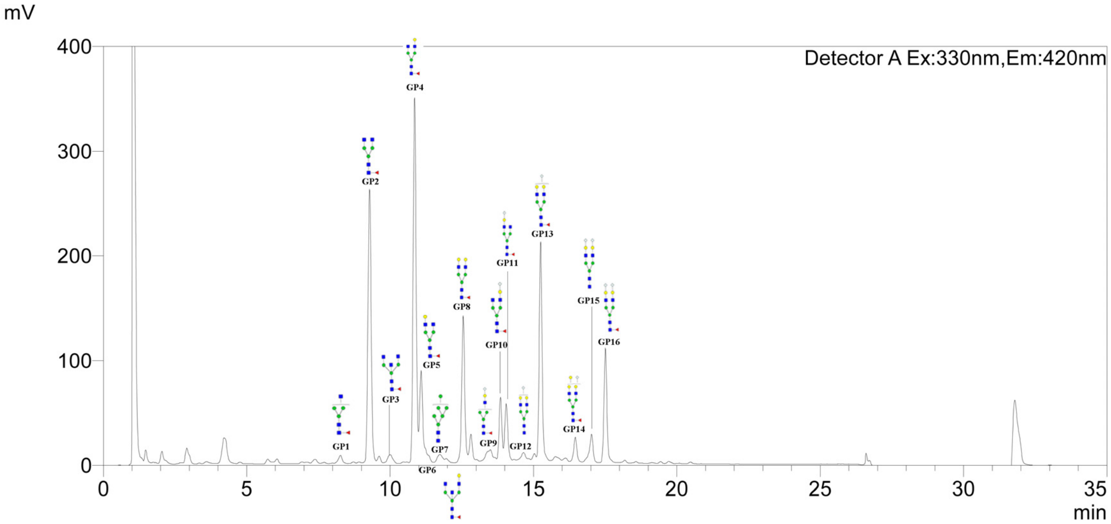

Figure S1.

Representative chromatogram of murine IgG N-glycans separated by UPLC-FLR after 2-AB labeling. The glycan structures of sixteen peaks (GP1-GP16) are presented. Structural symbols: blue square: N-acetylglucosamine; green circle: mannose; red triangle: fucose; yellow circle: galactose; white diamond: N-glycolylneuraminic acid.

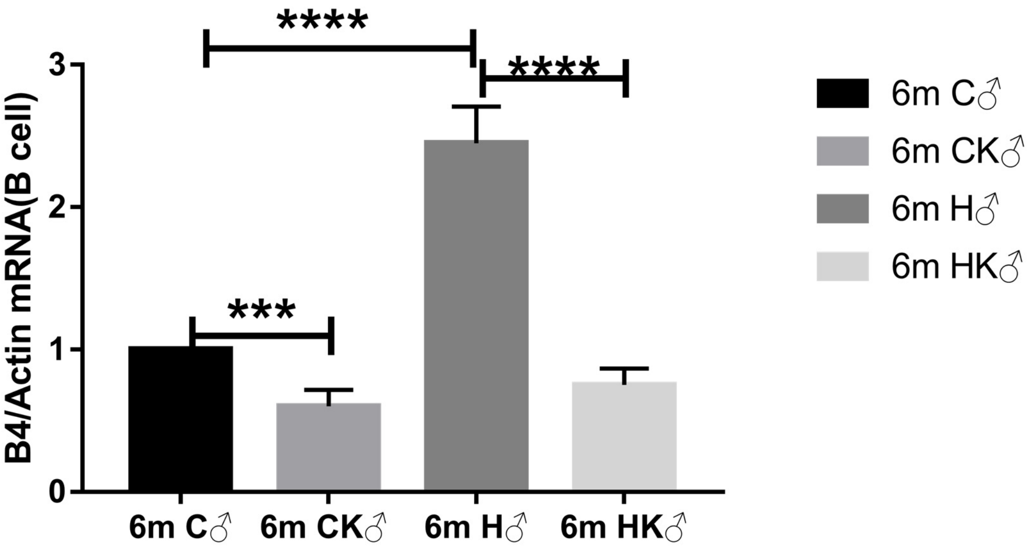

Figure S2

mRNA levels of B4GALT1 in spleen B cells of four mice in group C, group CK, group H, and group HK. According to the normalized statistics of mice in group C. Data are expressed as mean ±SD. ***$P < 0.001$, ****$P < 0.0001$. (C—control WT group; CK—control B4GALT1$^{+/-}$ group; H—HCC WT group; HK—HCC B4GALT1$^{+/-}$ group; 6m—6 months after administration; ♂—male mice).

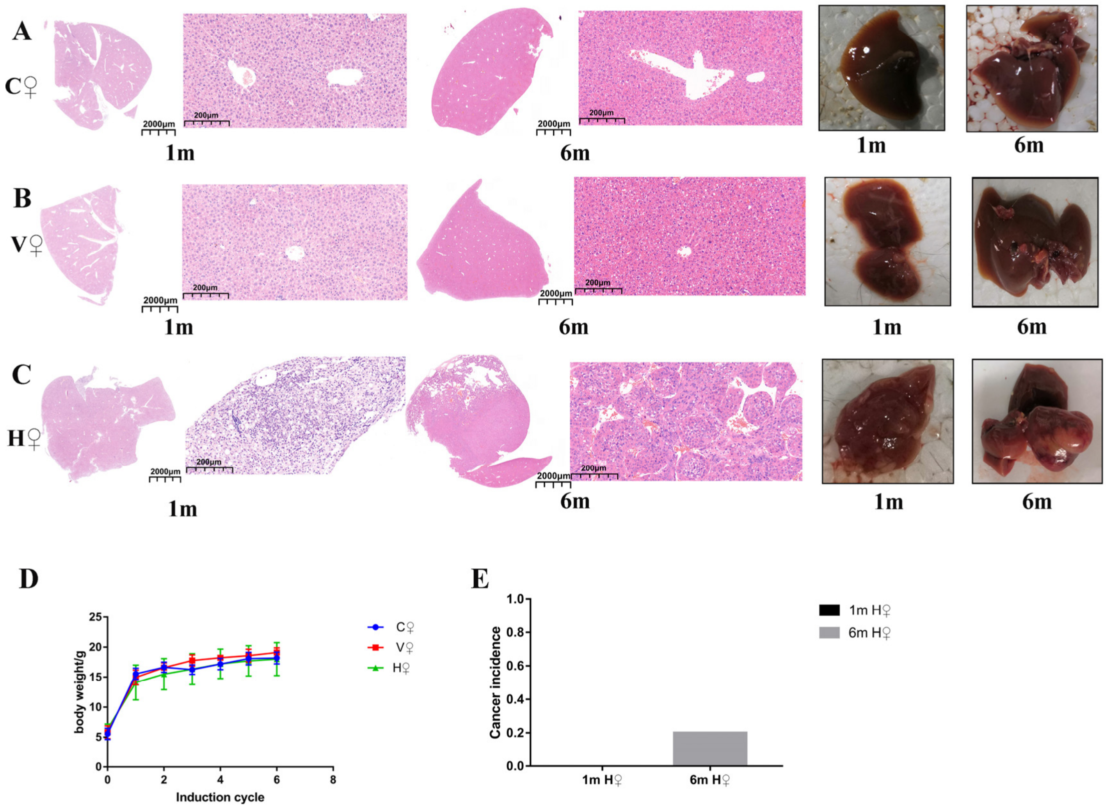

Figure S3.

DEN/CCl_4 induced HCC model was successfully established, and tumor formation was obvious after 6 months of administration. (A), Group C female mice showed no lesions in HE staining and visual observation 1 month and 6 months after administration **(B),** Group V female mice showed no lesions in HE staining and visual observation 1 month and 6 months after administration **(C),** Group H female mice developed vacuolar degeneration but did not form tumors 1 month after administration, and showed obvious tumor formation in HE staining and visual observation after 6 months. Hematoxylin and eosin, full scan image (×1.0), Bars=2000μm, single field image (×200), Bars=200μm. **(D),** There is no difference in the trend of body weight change in different groups of female mice during CCl_4 induction **(E),** Cancer incidence is 0 at 1 month after administration, and 20% at 6 months after administration. (C—control WT group; V—solvent WT group; H—HCC WT group; 1m/6m—1 month/6 months after administration; ♀—female mice).

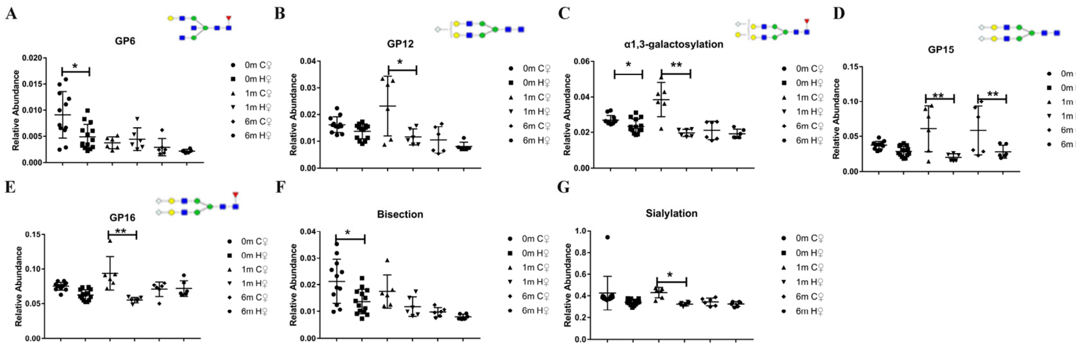

Figure S4

Female mice have no stable changes during the process of cancer induction. (A), The relative abundance of female mice GP6 in the process of cancer induction only decreased at 0 month **(B),** The relative abundance of GP12 in female mice only decreased at 1 month during the process of cancer induction **(C),** The relative abundance of α1,3-galactose in female mice decreases at 0 and 1 month during the process of cancer induction **(D),** The relative abundance of GP15 in female mice decreased at 1 and 6 months during the process of cancer induction **(E),** The relative abundance of GP16 in female mice only decreased at 1 month during the process of cancer induction **(F),** The relative abundance of female mice bisection during the induction of cancer only decreased at 0 month **(G),** The relative abundance of sialylation in female mice is only reduced at 1 month during the process of cancer induction**.** Structural symbols: blue square: N-acetylglucosamine; green circle: mannose; red triangle: fucose; yellow circle: galactose; white diamond: N-glycolylneuraminic acid. Data are expressed as mean ±SD. **P* < 0.05, ***P* <0.01, ****P* <0.001. (C—control WT group; H—HCC WT group; 0m/1m/6m—0 month/1 months/6 months after administration; ♀—female mice)

Table S1. The change trend of serum IgG N-glycan group H in female mice compared with group C at different time points in the process of cancer induction.

Glycan peak	0 month	1month	6month	8month	Component name
GP1	_	_	_	_	F(6)A1
GP2	_	_	_	_	F(6)A2
GP3	_	_	_	_	F(6)A2B
GP4+5	↑↑	↑↑↑	_	_	F(6)A2G1
GP6	↓	_	_	_	F(6)A2[6]BG1
GP7	_	_	_	_	M6
GP8	_	↑↑↑	_	_	F(6)A2G2
GP9	_	_	_	_	F(6)A1G1S1
GP10	_	_	_	_	F(6)A2[6]G1S1
GP11	_	_	_	_	F(6)A2[3]G1S1
GP12	_	↓	_	_	A2G2S1
GP13	_	_	_	_	F(6)A2G2S1
GP14	↓	↓↓	_	_	F(6)A2G2G(3)1S1
GP15	_	↓↓	↓↓	_	A2G2S2
GP16	_	↓↓	_	_	F(6)A2G2S2
Galactosylation	_	_	_	_	
Fucosylation	_	↑↑	_	_	
Sialylation	_	↓	_	_	
Bisection	↓	_	_	_	
Monoantennary glycans	_	_	_	_	

Footnote: ↑&↓ represents $P < 0.05$, ↑↑&↓↓ represents $P < 0.01$, ↑↑↑&↓↓↓ represents $P < 0.001$.

Method about UPLC-FLR

After the glycan samples were spin dried and resolved, the glycan solution was analyzed by UPLC. We measured the relative abundance of each individual peak by dividing its area by the total area of 16 peaks. The labeled N-glycans were separated by UPLC LC-30A (Shimadzu) with a fluorescence detector set at excitation and emission wavelengths of 330 and 420 nm, respectively. UPLC separation was performed with ACQUITY UPLC BEH Amide columns (2.1 × 150 mm, 1.7 μm; Waters, USA). The stationary phase was gradient eluted with 100 mM ammonium formate (solvent A)/acetonitrile (solvent B; A: 50%–25% / B: 50%–75%). The flow rate, column temperature, and injection volume were 0.4 mL min^{-1}, 60°C, and 2μL, respectively. The instrument was controlled using LabSolution software(Shimadzu)[1].

1. Gu, Y.; Han, J.; Liu, X.; Pan, Y.; Xu, X.; Sha, J.; Ren, S.; Gu, J., Dynamic alterations in serum IgG N-glycan profiles in the development of colitis-associated colon Cancer in mouse model. *Biochim Biophys Acta Gen Subj* **2020,** 1864, (10), 129668.
